# Discovery of a Novel Leaf Rust (*Puccinia recondita*) Resistance Gene in Rye (*Secale cereale* L.) Using Association Genomics

**DOI:** 10.3390/cells11010064

**Published:** 2021-12-27

**Authors:** Nikolaj Meisner Vendelbo, Khalid Mahmood, Pernille Sarup, Mogens S. Hovmøller, Annemarie Fejer Justesen, Peter Skov Kristensen, Jihad Orabi, Ahmed Jahoor

**Affiliations:** 1 Nordic Seed A/S, Grindsnabevej 25, 8300 Odder, Denmark; khma@nordicsee.com (K.M.); pesa@nordicseed.com (P.S.); pskr@nordicseed.com (P.S.K.); jior@nordicseed.com (J.O.); ahja@nordicseed.com (A.J.); 2 Department of Agroecology, Faculty of Technical Sciences, Aarhus University, 4200 Slagelse, Denmark; mogens.hovmoller@agro.au.dk (M.S.H.); annemariefejer.justesen@agro.au.dk (A.F.J.); 3 Department of Plant Breeding, The Swedish University of Agricultural Sciences, 23053 Alnarp, Sweden

**Keywords:** hybrid breeding, Gülzow germplasm, field trial, disease progression, 600K high-density SNP array, nucleotide-binding leucine-rich repeat (NLR), NB-ARC domain, phylogenetic analysis, *Lr1*-like disease resistance protein

## Abstract

Leaf rust constitutes one of the most important foliar diseases in rye (*Secale cereale* L.). To discover new sources of resistance, we phenotyped 180 lines belonging to a less well-characterized Gülzow germplasm at three field trial locations in Denmark and Northern Germany in 2018 and 2019. We observed lines with high leaf rust resistance efficacy at all locations in both years. A genome-wide association study using 261,406 informative single-nucleotide polymorphisms revealed two genomic regions associated with resistance on chromosome arms 1RS and 7RS, respectively. The most resistance-associated marker on chromosome arm 1RS physically co-localized with molecular markers delimiting *Pr3*. In the reference genomes Lo7 and Weining, the genomic region associated with resistance on chromosome arm 7RS contained a large number of nucleotide-binding leucine-rich repeat (NLR) genes. Residing in close proximity to the most resistance-associated marker, we identified a cluster of NLRs exhibiting close protein sequence similarity with the wheat leaf rust *Lr1* gene situated on chromosome arm 5DL in wheat, which is syntenic to chromosome arm 7RS in rye. Due to the close proximity to the most resistance-associated marker, our findings suggest that the considered leaf rust *R* gene, provisionally denoted *Pr6*, could be a *Lr1* ortholog in rye.

## 1. Introduction

Winter rye (*Secale cereale* L.) is an important cereal crop in Northern and Eastern Europe, with an acreage of 903,800 ha in Poland, 636,300 ha in Germany, 146,600 ha in Denmark and 113,200 ha in the Baltic countries in 2017 [1]. Since the introduction of hybrids in the mid-1980s, the primary focus of resistance breeding in rye shifted towards ergot caused by *Claviceps purpurea* (Fr.) Tul [2,3]. Recent decades of breeding efforts and the introduction of the exotic restoration of male fertility genes have, however, reduced the importance of ergot and the resulting toxic alkaloids in rye grains [4,5]. This provides an opportunity to focus on leaf rust, also termed brown rust, which has received little scientific attention and commercial interest in recent years. In 2002, Miedaner et al. [6] reported a low level of inherent resistance to leaf rust in the predominant hybrid rye breeding gene pools Petkus and Carsten. This is still the case based on observations of top-yielding hybrid cultivars and population varieties in the Danish official trials, which showed 9.5% mean leaf rust severity in 2019 (Table S2 [7]). Similarly, the German list of recommended varieties of rye had 11.5% mean leaf rust severity in 2021 (Table S2 [8]). Under moderate to severe infection levels, leaf rust has been reported to cause a 11–27% reduction in grain yield, in addition to considerable quality losses [9].

The cereal rusts are heteroecious, requiring two taxonomically unrelated host plants to complete their life cycle, and macrocyclic, entailing five distinct spore types corresponding to different life stages [10,11,12]. During the growing season, rusts can cause severe epidemics through repetitive infection and clonal reproduction of asexual urediniospores [13,14]. As obligate biotrophs, rusts require a living host for reproduction, but it may survive parts of the year as telia on rye plant debris, or as aecia on the alternate host [15]. In years with conditions conducive to leaf rust, the successive autumn-sown winter rye crop can likewise be infected, leading to an early establishment [16].

Cereal rusts are capable of migrating long distances by wind dissemination of urediniospores [17,18,19,20], which may result in exotic incursions of new races from distant areas [21]. In wheat, two new races of yellow rust, ‘Warrior’ (Pst7) and ‘Kranich’ (Pst8), first detected in Europe in 2011, are believed to have migrated from a sexual population in the near-Himalayan region of Asia [22]. Sexual recombination in rust fungi facilitates the development of new virulence combinations, driving adaptation to deployed host resistance and the emergence of novel aggressive pathotypes [22,23]. Population studies on wheat leaf and yellow rust populations, however, reveal clonal population structures and the absence of sexual recombination in many areas [24,25,26,27]. In such populations, novel genetic variation may be driven by means of mutations, somatic hybridization and internuclear exchange [23,28].

In rye, leaf rust is caused by a fungal basidiomycete *Puccinia recondita* f. sp. *secalis* Roberge. ex Desmaz. (*Prs*) [29]. Unlike the alternate hosts for the rusts of wheat, the alternate host of *Prs*, small bugloss (*Anchusa arvensis* L.), is widespread in the North European flora, being a common weed in agricultural fields [12,30,31,32]. Observation of high pathotype diversity and virulence complexity in the German *Prs* population may suggest the occurrence of sexual recombination in rye leaf rust [33], which may facilitate *Prs* to overcome deployed resistance (*R*) genes [23,33].

Currently, five major leaf rust *R* genes have been identified in rye, *Pr3* (1RS), *Pr4* (1RL), *Pr5* (1RL), *Pr1* (6RL) and *Pr2* (7RL) [34,35]. An additional three major leaf rust *R* genes have been identified in wheat–rye substitution and translocation lines, in the wheat gene nomenclature denoted *Lr26* (1BL-1RS), *Lr25* (4BS.4BL-2RL) and *Lr45* (2AS-2RS.2RL) [36,37].

Most major *R* genes belong to a large family of nucleotide-binding leucine-rich repeat proteins (NLR) [38]. In rye, 1,167 NLR genes have been identified in the Lo7 reference genome and 1,447 NLR genes in the Weining reference genome [39,40]. In grasses, the canonical NLR gene consists of three domains: a C-terminus leucine-rich repeat (LRR) domain, involved in pathogen effector recognition [41], a central nucleotide-binding (NB) domain functioning as a regulatory domain determining protein activation state [42] and an N-terminus coiled-coil (CC) domain believed to be involved in signaling and the induction of cell death [43].

Genomic-based breeding techniques have accelerated the introgression and pyramiding of *R* genes for enhancing resistance durability [44,45,46]. Recent advances in genomic resources available in rye, including the 600K high-density SNP array and chromosomal-scale reference genomes of a German inbred winter rye line Lo7, and a Chinese population rye variety Weining, respectively, constitute significant milestones in rye genomic breeding [39,40,47]. In order to expand the ‘toolbox’ available for resistance breeding in rye, continuous mining for the discovery of novel genetic variability in *R* genes is essential.

In this paper, we investigate leaf rust resistance in a Gülzow-based elite hybrid rye breeding germplasm. The Gülzow germplasm is differentiated from the predominant hybrid rye breeding gene pools, Petkus and Carsten, by a distinct cytoplasmic male sterility system [48,49]. Our objectives were to (I) characterize leaf rust resistance and disease progression in the assayed germplasm, (II) identify genomic regions and molecular markers associated with leaf rust resistance to facilitate marker-assisted selection for leaf rust resistance and (III) investigate in silico whether NLR genes residing in leaf rust resistance-associated regions on the Lo7 and Weining reference genomes resemble known leaf rust *R* genes.

## 2. Materials and Methods

### 2.1. Plant Material and DNA Extraction

A panel of 180 inbred rye (*Secale cereale* L.) lines, 92 restorer and 88 non-restorer germplasms, belonging to the Gülzow-based elite hybrid rye breeding germplasm at Nordic Seed A/S (Dyngby, Denmark), were investigated in this study. Population structure and information on the genetic characteristics of the accessions were investigated in a recent study by Vendelbo et al. [50]. DNA extraction was done using an adapted SDS-based method according to USDA [51], after Pallotta et al. [52], on an equivalent of 75 mg plant material collected from the primary leaves of two seven-day old seedlings per line. DNA concentration and 260/280 nm absorption ratio of samples were measured using an Epoch^TM^ microplate spectrophotometer (Biotek^®^, Santa Clara, CA, USA) and evidence of fragmentation by size visualization on 1.2% agarose gel.

### 2.2. Molecular Marker Resource and SNP Genotyping

Samples of each line containing 200 ng high-molecular-weight gDNA with ≥1.8 260/280nm ratio were sent for single-nucleotide polymorphism (SNP) genotyping at Eurofins Genomics Europe Genotyping (Aarhus, Denmark). Genotyping was done using a 600K SNP array with 600,843 SNP markers on a Affymetrix GeneTitan^TM^ Scanner platform (Thermo Fisher Scientific Inc., Waltham, MA, USA) [47]. Marker map for the 600K SNP array on the Lo7 reference genome and evaluation of its performance in the assayed germplasm was recently presented in a study by Vendelbo et al. [53]. The marker map was acquired from https://doi.org/10.5281/ZENODO.5086235 (Access date: 9 July 2021). Prior to analysis, markers were filtered for marker allele frequency ≥0.05, missing individual score ≤ 0.2 and missing marker score ≤ 0.1 to identify informative markers.

### 2.3. Collection of Puccinia Recondite f. sp. secalis Populations

To establish a representative bulk inoculum of *Puccinia recondita* f. sp. *secalis* (*Prs*) for inoculation of field trials, leaf samples of rye in the field were collected from four locations in the period May-July 2018 in Denmark and Northern Germany. In Denmark, the locations were Dyngby in Jutland (55.9479° N, 10.2572° E), Flakkebjerg on Zealand (55.3255° N, 11.3826° E) and Holeby on Lolland (54.6998° N, 11.4511° E). In Northern Germany, the location was Nienstädt (52.3451° N, 9.1664° E). Multiplication of *Prs* was done at the Global Rust Reference Centre (GRRC), Institute of Agroecology, Aarhus University (Flakkebjerg, Denmark). The detailed protocol has been provided at https://doi.org/10.5281/zenodo.5478060 (Access date: 9 July 2021). Spores from each leaf sample were multiplied individually, resulting in 10 ‘unique’ *Prs* field populations from each of the four sampled locations. For multiplication of *Prs*, a 50:50 mixture of hybrid rye cvs. KWS Bono and KWS Florano was selected on the basis of high leaf rust susceptibility reported in the Danish official trials from 2015 to 2018 [7].

### 2.4. Field Trial

Field trials were conducted at three locations, two situated in Denmark at Gylling in Central Jutland (55.8946° N, 10.1705° E) and Flakkebjerg on Zealand (55.3216° N, 11.3901° E), and one located in Northern Germany at Nienstädt in Niedersachsen (52.3556° N, 9.2270° E). Trials were sown in a Seedmatic^®^ layout with a single parcel (1.0 m × 1.25 m) consisting of six rows of approximately 25 plants per breeding line, with a between-row distance of 25 cm and between-parcel distance of 40 cm. Each block comprised 18 parcels, with three blocks per replicate, of which the first was laid down in numerical order and the second in an incomplete randomized block design [54]. Hybrid cv. KWS Serafino was included as a ‘resistant’ control, and cv. KWS Binntto as ‘susceptible’, selected on the basis of disease severity recordings in the Danish official trials from 2015 to 2018 [7]. At Nienstädt and Flakkebjerg, a leaf-rust-susceptible spreader row consisting of 50:50 hybrid cvs. KWS Binntto and KWS Serafino was sown as a spreader row at 2nd and 4th row position in each parcel to facilitate artificial inoculation. The trial site at Gylling was not artificially inoculated and no spreader rows were sown here. The Gylling trial site was sown on the 18th of September 2018, Flakkebjerg the 25th of September and Nienstädt the 4th of October in 2018. The trial at Nienstädt was repeated and sown on the 13th of October 2019.

### 2.5. Field Inoculation

Flakkebjerg and Nienstädt were artificially inoculated using seedlings with sporulating rust that had been inoculated with *Prs* field populations collected in 2018. The Gylling trial site in 2019 was kept for natural infection to study the progression of leaf rust disease under non-inoculated conditions. For each of the two sites, four sowing trays were prepared, each containing 35 multiplication pots. Multiplication of field trial inoculum was done according to the protocol provided at https://doi.org/10.5281/zenodo.5478060 (Access date: 9 July 2021). Inoculation of trays was done using an airbrush compression system in accordance with Thach et al. [27]. A spore inoculum solution was prepared for each of the trials in Denmark by mixing one tube, containing ≈ 6–7 mg spores, for each of the 30 *Prs* field populations collected at the three Danish locations in 2018 in a single tube by shaking for 60 s. The spore inoculum was split into four equal portions, one per tray, of approximately 50 mg, transferred to a 50 mL airbrush glass container and suspended in 4 mL 3MTM NovecTM 7100 engineering fluid. The inoculum solution for the trial in Northern Germany was prepared following the same procedure, using the *Prs* populations collected at Nienstädt. At 14 DAI, rusts were sporulating on the seedlings and trials were inoculated by brushing a single multiplication pot over three consecutive spreader rows. Three days later, multiplication pots were brushed in a similar manner across the same spreader rows in the opposite direction, plastic pots removed and seedlings planted. Trials were artificially inoculated in April in both years, following the same procedure to ensure uniform disease pressure.

### 2.6. Disease Scoring

Trials were scored for leaf rust severity a minimum of two times using a 1–9 scoring scale (Supplementary Table S1, [55]). The lines were scored by evaluating all plants of the individual breeding line within the plot. The experiment at Gylling, Denmark was scored four times in 2019 from the first detection of leaf rust in May until crop senescence at the end of June to study the disease progression under untreated conditions.

### 2.7. Analysis of Disease Scoring Data

Area under disease progress curve (AUDPC) was calculated at the non-treated site, Gylling, Denmark, using the agrocolae (v. 1.3–5) package in R [56]. For the purpose of interpretation, disease severity scale was adjusted to calculate a corrected AUDPC starting from zero (completely resistant with no evidence of chlorosis). Based on AUDPC and the disease progression curve at Gylling, lines were placed into four groups in order to characterize the assayed germplasms’ qualitative and quantitative resistance to leaf rust. The groups comprised (i) resistant (‘R’), with an AUDPC less than 20, (ii) partially resistant (‘P–R’), with an AUDPC of 20–80, (iii) partially susceptible (‘P–S’) and (iv) susceptible (‘S’), with an AUDPC between 70 and 170. While the AUDPC intervals of groups overlapped, each was distinguished by their disease progression; breeding lines assigned to (i) ‘R’ had a near-linear curve with disease severity less than 2, (ii) ‘PS’ had a stable inclining curve with a terminal severity score less than 6, (iii) ‘P–S’ had a late occurrence with a terminal severity score between 6 and 8, and (iv) ‘S’ had an early occurrence with a terminal severity score between 6 and 8. A pairwise t-test using a standard Bonferroni correction for multiple comparison was done on the four groups’ AUDPC scores using inherent functions in R.

In order to correct the resistance phenotype for effects of replicate, block position, population, location-year and *G* × *E* interaction effect, four linear mixed models were constructed using the lme4 (v. 1.1.26) package in R. The models were used for (1) individual locations per parental population, (2) individual locations using the entire assayed germplasm, (3) all locations per parental population and (4) all locations using the entire assayed germplasm

y = µ + b + r + G + ε

y = µ + b + r + P + G + ε

y = µ + b + r + G + E + G×E + ε

y = µ + b + r + P + G + E + G × E + ε

where *µ* is the general mean, *b* is the block, *r* is the replicate, *P* is the population, *G* is the line id, *E* is the location-year, *G* × *E* is the genotype, i.e., line and environment interaction effect, and *ε* is the residuals. The parameters *b*, *r* and *G* × *E* were set as random effects and *P*, *E* and *G* were set as fixed effects. The random effects and residuals were assumed to be independent, normally distributed variables described as follows: *b*~N (0, I $\sigma_{b}^{2}$), *r*~N (0, I $\sigma_{r}^{2}$), *G × E*~N (0, I $\sigma_{G\times E}^{2}$) and *ε*~N (0, I $\sigma_{\varepsilon }^{2}$). The best linear unbiased estimator (BLUE) for the line effect, referred to as the resistance value, was used as phenotypic input for GWAS.

The broad sense heritability per plot (*H*^2^) was extracted using a modified model no. 4 with line set as random effect, distributed *G*~N (0, I $\sigma_{G}^{2}$) and estimated as

$$H^{2}\;=\;\frac{\sigma_{G}^{2}}{\sigma_{G}^{2}+\sigma_{b}^{2}+\sigma_{r}^{2}+\sigma_{G\times E}^{2}+\sigma_{\varepsilon }^{2}}$$

where $\sigma_{l}^{2}$ is line variance, $\sigma_{b}^{2}$ is block variance, $\sigma_{r}^{2}$ is replicate variance, $\sigma_{G\times E}^{2}$ is genotype–environment interaction variance, and $\sigma_{\varepsilon }^{2}$ is the residual variance.

### 2.8. Genome-Wide Association Study

Discovery of leaf rust resistance-associated SNP markers was done by genome-wide association study (GWAS) using the genomic association and prediction integration tool (GAPIT) (v.3) package in R [57]. The Manhattan plot was colorized using the RColorBrewer (v.1.1-2) R package colour palette [58]. GWAS was done using both the mixed linear model (MLM) and the Bayesian information and Linkage Disequilibrium Iteratively Nested Keyway (BLINK) method [59]. BLINK uses a multiple loci test for MLM by combining a fixed effects model, Bayesian information content and linkage disequilibrium information, collectively improving the statistical power in the GWAS. Markers that are in linkage disequilibrium with the most significant marker at a site are excluded in BLINK. A standard Bonferroni-corrected threshold of α = 0.05 was used as the significance threshold. To investigate whether identified resistance-associated SNP markers on chromosome arm 1RS resided in proximity to known *Pr* genes (*Pr3*, *Pr4*, *Pr5*), flanking co-segregating markers were extracted and anchored to the Lo7 and Weining reference genomes using the NCBI blast function [35,60,61].

### 2.9. Phylogenetic Analysis of Lines

In order to investigate the phylogenetic distribution of resistance, a neighbor-joining clustering analysis of breeding lines was done using available SNP marker data with the Euclidean genetic distance measure in the ape (v. 5.3) R package [62]. The tree was constructed after 10.000 bootstrapping iterations, with weak nodes (≤80% recurrence) collapsed into multifurcations. Circular neighbor-joining tree was generated using the iTOL (v. 5) online tool (http://itol.embl.de/, access date: 5 October 2021), enabling a color visualization of the resistance response of each line at the three field trial locations and the distribution of lines carrying the resistance concentric circles [63].

### 2.10. Phylogenetic Analysis and In Silico Characterization of Nucleotide-Binding Leucine-Rich Repeat Genes in Leaf Rust Resistance-Associated Regions

The leaf rust resistance-associated sites in the Weining reference genome were identified by the mapping of resistance-associated markers using the same procedure as described previously for the Lo7 reference genome. Nucleotide-binding leucine-rich repeat (NLR) genes residing in leaf rust resistance-associated regions in the Lo7 and Weining reference genomes were identified using the NLR annotation provided in a recent study by Vendelbo et al. [53], available at https://doi.org/10.5281/zenodo.5085854 (Access date: 9 July 2021). Coding sequences of potential candidate NLR genes were extracted using an online data repository [39,40]. Gene structures of NLR genes were predicted using the AUGUSTUS (3.4.0) program [64].

To investigate whether NLR genes residing in leaf rust resistance-associated sites on the Lo7 and Weining reference genomes resembled known leaf rust *R* genes, the NCBI blastp function was used for a protein–protein search in the online database and a phylogenetic analysis was conducted using a panel of cloned cereal rust *R* genes as reference [61]. The phylogenetic analysis was performed using NLR genes’ conserved NB-ARC (nucleotide-binding adaptor shared by APAF-1, R proteins and CED-4) domain sequences. The NB-ARC sequences of NLR genes residing within the sites on the Lo7 and Weining reference genomes were extracted from the data repository file referred to above by Vendelbo et al. [53]. The panel of known NLR-type *R* genes comprised leaf rust (*Lr1*, *Lr10*, *Lr21*, *Lr22a*), stem rust (*Sr13*, *Sr22*) and yellow rust (*Yr5*, *Yr10*, *Yr28*). NB-ARC sequences of reference NLR genes were obtained from the UniProt online database [65]. Phylogenetic analysis was conducted using a pipeline developed by Toparslan et al. [66] in R. Multiple sequence alignment of NB-ARC domain sequences was done using the msa (v. 1.20.1) and pairwise genetic distance based on identity calculated using the seqinr (4.2-8) package in R [67,68]. A tree was constructed for the respective NLR repertoire of Lo7 and Weining reference genome and visualized using the ggtree (v. 2.2.4) R package [69].

### 2.11. Graphical Editing

Graphs and figures were outputted from R in svg format and manually curated using the Inkscape (v. 1.1) program (https://inkscape.org/, accessed on 7 September 2021).

## 3. Results

### 3.1. 600 K SNP Genotyping of Panel

Quality filtration of markers for low minor allele frequency, missing markers and missing individual scores across the panel led to the identification of 261,406 informative markers. Markers were homogeneously distributed across the rye genome, with an average of 32,676 markers per chromosome and mean marker-to-marker distance of 25.54 kb.

### 3.2. Phenotyping of Leaf Rust Resistance

**Figure 1:**
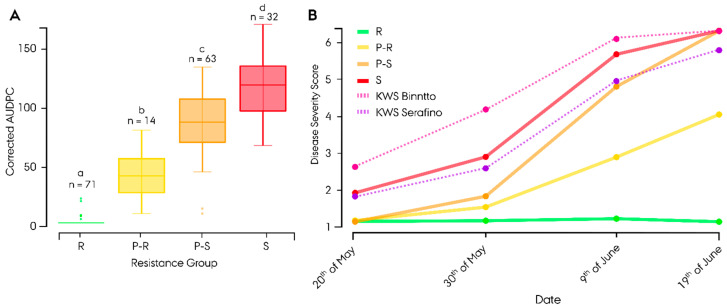
Leaf rust disease severity in 180 hybrid rye (*Secale cereale* L.) breeding lines grouped according to resistance response at Gylling (Denmark) field trial in 2019. (**A**) Area under disease progress curve (AUDPC) boxplot with standard error of each assigned resistance response group—resistant (‘R’), partially resistant (‘P-R’), partially susceptible (‘P-S’) and susceptible (‘S’) groups—with different letters indicating significant differences (*p* < 0.05). (**B**) Mean disease progression curve of each group during the growing season, including resistant control hybrid cv. KWS Binntto and susceptible control hybrid cv. KWS Serafino.

In both 2019 and 2020, field trials demonstrated a high level of leaf rust disease, with a clear segregation of resistance within the assayed germplasm. Based on the AUDPC and disease progression at the natural infection site, Gylling, lines were divided into four groups (Supplementary Tables S3 and S5). The resistant (‘R’) group consisted of 48 restorer and 23 non-restorer germplasm lines, displaying a mean final disease severity (1–9) of 1.38 ± 0.84 standard deviation (SD) across trials (Supplementary Table S5). The partially resistant (‘P-R’) group consisted of nine restorer and five non-restorer germplasm lines, displaying a mean final disease severity of 3.85 ± 2.29 SD across trials. The partially susceptible (‘P-S’) group consisted of 12 restorer and 52 non-restorer germplasm lines, displaying a mean final disease severity of 7.21 ± 1.47 SD across trials. Finally, the susceptible (‘S’) group consisted of 23 restorer and 8 non-restorer germplasm lines, displaying a mean final disease severity of 7.21 ± 1.34 SD across year-location. AUDPCs for each group were 0.97 ± 3.6 SD for the ‘R’ group, 41.4 ± 22.9 SD for the ‘P-R’ group, 84.6 ± 25.8 SD of the ‘P-S’ group and 116.4 ± 26.6 SD for the ‘S’ group (Figure 1A). Calculation of a pairwise t-test showed that the AUDPC distribution of all groups differed significantly (*p* < 0.05). Susceptible control hybrid cv. KWS Binntto demonstrated a mean final disease severity of 7.29 ± 0.86 SD across trials, with an AUDPC of 144.1 (Supplementary Table S4, Figure 1B). Resistant control hybrid cv. KWS Serafino demonstrated a mean final disease severity of 6.75 ± 1.03 SD across trials, with an AUDPC of 91.3. 

**Figure 2:**
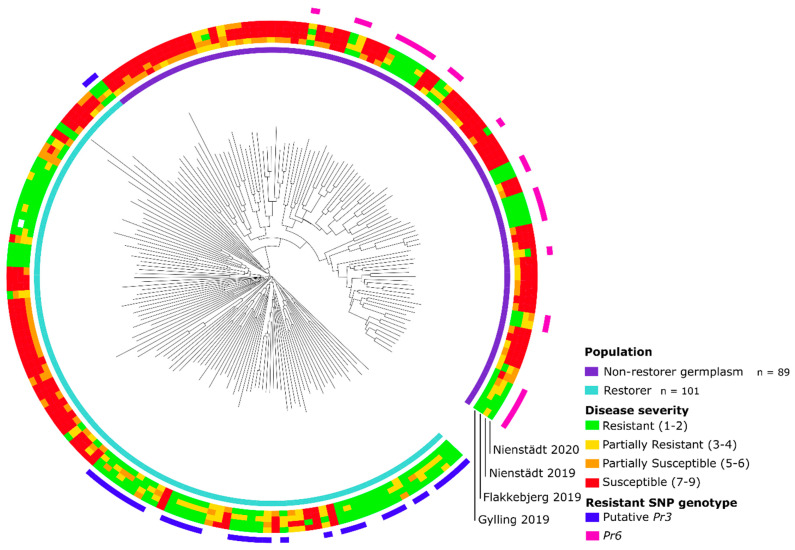
Circular neighbor-joining dendrogram of 180 hybrid rye (*Secale cereale* L.) breeding lines using 261,406 informative SNP markers. Leaf rust resistance (1–9) at four field trials in Denmark and Northern Germany in 2019 and 2020 displayed by concentric circles around the dendrogram. Lines carrying the resistant genotype of single-nucleotide polymorphism (SNP) markers associated with leaf rust resistance gene on chromosome arm 1RS (putative *Pr3*) and 7RS (*Pr6*) are likewise displayed in the concentric circles.

The resistance spectrum of breeding lines was visualized in a circular neighbor-joining dendrogram in iTOL, with concentric circles added to integrate the phenotypic score at each trial site (Figure 2). The phylogenetic analysis revealed a uniform distribution of resistant lines across the tree in both parental populations, while the majority of susceptible lines in the restorer population were largely found to form a secluded clade.

### 3.3. Genome-Wide Association Study

**Figure 3:**
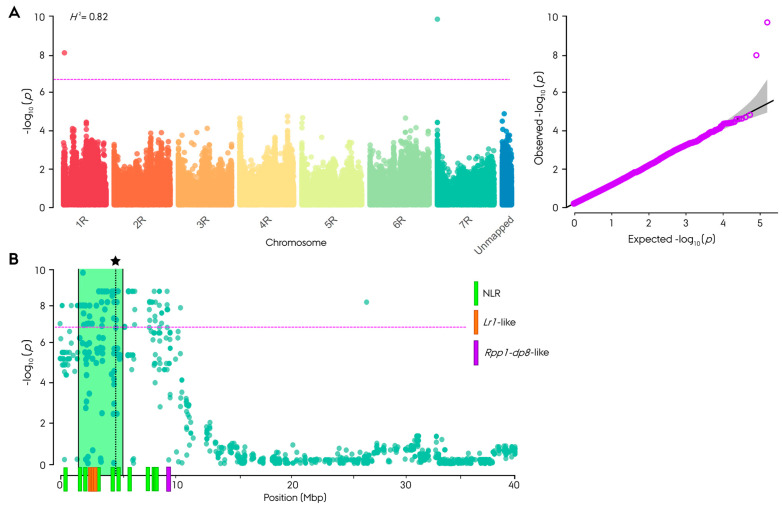
Manhattan plot for genome-wide association study (GWAS) using BLINK method on leaf rust disease resistance in 180 hybrid rye (*Secale cereale* L.) breeding lines using 261,406 informative SNP markers anchored to the Lo7 reference genome. (**A**) Using best linear unbiased estimator (BLUE) resistance value across four field trials in 2019 and 2020 for the entire germplasm as phenotypic input, including Q-Q plot. (**B**) Excerpt of the chromosome arm 7RS from GWAS using the same phenotypic input as in (**A**) with MLM method instead. The span of most associated marker position is highlighted in green, mean position of most associated marker by an asterisk and NLR genes in the Lo7 reference genome by vertical bars. The purple line represents the Bonferroni-adjusted significance threshold based on informative markers.

For the identification of SNP markers associated with leaf rust resistance, a genome-wide association study (GWAS) was done on each of the individual field trial locations, across all trials and on AUDPC using both the entire germplasm and individual parental populations (Supplementary Figure S1). GWAS conducted on the entire germplasm and non-restorer germplasm population led to a consistent finding of a highly significant peak (−log_10_(*p*) = 9.7–48.5) on chromosome arm 7RS from 1.56 to 4.85 Mb, with the most associated marker at 4.7 Mb (Figure 3A, Supplementary Table S6). The most leaf rust resistance-associated SNP markers on chromosome arm 7RS explained between 15.9 and 27.1% of the phenotypic variance (Supplementary Table S6). In the GWAS conducted on the entire population across all trials, a significant peak (−log_10_(*p*) = 7.9) was identified at 115.1 Mb on chromosome arm 1RS, explaining 11.8% of the phenotypic variance (Figure 3A). The *SCM9* and *Xscm1* markers co-segregating with *Pr3*, and flanking either side of the gene, mapped to 96.7 and 137.6 Mb on chromosome arm 1RS of Lo7 and 118.1 to 184.1 Mb, respectively, on the Weining reference genome. None of the GWAS analyses conducted on the restorer population alone led to the observation of significant associated SNP markers (Supplementary Figure S1). Non-significant peaks were, however, observed on chromosome arms 1RS, 2RS, 2RL 3RS, 6RL, 7RS and 7RL in the restorer population, explaining between 6.9 and 10.2% of the phenotypic variance. While additional markers with a moderately significant association were identified in GWAS on the entire germplasm and non-restorer germplasm, these were disregarded due to inadequate consistency in the discovery relationship. Using MLM-GWAS, the leaf rust resistance-associated region on chromosome arm 7RS was found to span 11 Mb from the distal tip (Figure 3B).

The resistant allele of the most resistance-associated marker, AX-99370891, on chromosome arm 7RS was exclusive to the non-restorer germplasm population and found in all 23 resistant lines, two out of nine partially resistant lines and one susceptible line (Figure 2, Supplementary Table S6). The resistant allele of the most resistance-associated marker, AX-99442596, on chromosome arm 1RS was exclusive to the restorer population and found in 33 out of 48 resistant lines and two out of nine partially resistant lines (Figure 2, Supplementary Table S6).

The plot-based broad sense heritability (*H*^2^) was high for all of the linear mixed models conducted both on individual and across parental populations and locations, ranging from 0.79 to 0.92 (Supplementary Figure S1, Supplementary Table S7). In the non-restorer population, the *H*^2^ ranged from 0.82 to 0.92, and in the restorer population, the *H*^2^ ranged from 0.79 to 0.87.

### 3.4. Phylogenetic Analysis and In Silico Characterization of Nucleotide-Binding Leucine-Rich Repeat Genes in Leaf Rust Resistance-Associated Block on Chromosome Arm 7RS

Mapping of 442 out of 685 resistance-associated markers on chromosome arm 7RS in the Lo7 reference genome to the Weining reference genome led to the positioning of the resistance-associated region from 5.8 to 23.6 Mb on chromosome arm 7RS (Supplementary Table S8). Gene mining in the resistance-associated region led to the identification of 33 nucleotide-binding leucine-rich repeat (NLR) genes in the Lo7 reference genome and 38 NLR genes in the Weining reference genome (Supplementary Table S9).

**Figure 4:**
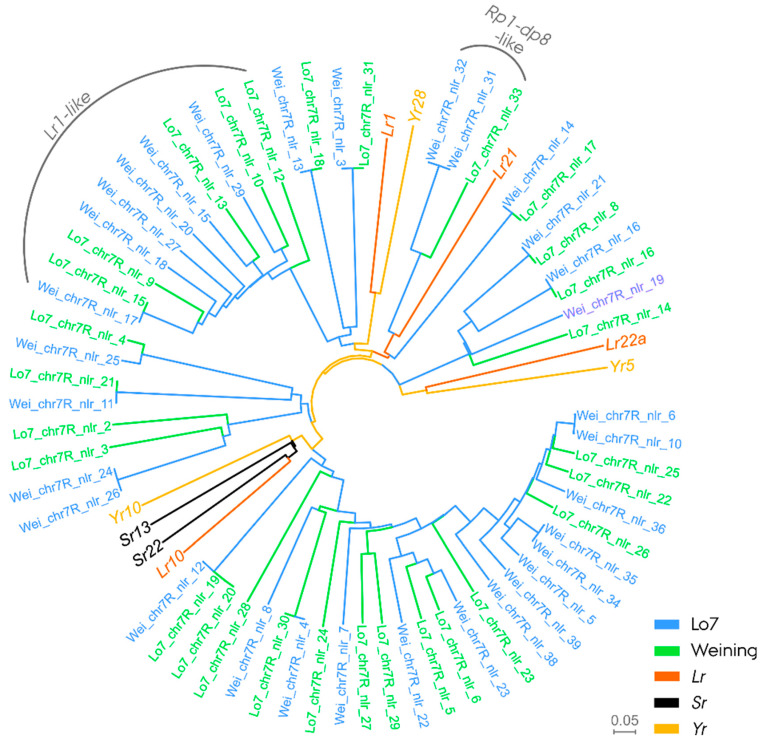
Phylogenetic relationship of nucleotide-binding leucine-rich repeat (NLR) genes in a leaf rust resistance association region on rye (*Secale cereale* L.) chromosome arm 7RS in the reference genomes Lo7 and Weining. The tree was constructed using the central NB-ARC domain sequence. Panels of known wheat leaf rust (*Lr*), stripe rust (*Yr*) and stem rust (*Sr*) genes are included as reference.

Phylogenetic analysis and in silico characterization led to the identification of a large cluster of full-length (‘complete’) NLR genes in both reference genomes, showing ≤80.7% sequence similarity at a complete alignment with wheat leaf rust *R* gene Lr1 (Figure 4, Table 1 and Table S10). In the Lo7 reference genome, the cluster consisted of five NLR genes situated at 2.7 to 2.9 Mb, with the most leaf rust resistance-associated markers from 1.59 to 4.9 Mb (Supplementary Table S6). In the Weining reference genome, the cluster consisted of six NLR genes situated at 12.2 to 14.5 Mb, with the most leaf rust resistance-associated markers positioned from 10.0 to 12.8 Mb (Supplementary Table S6). The resistance-associated region on chromosome arm 7RS also housed NLR genes in both reference genomes, showing ≤ 59.2% sequence similarity, aligning from 350 to 534 of the entire 1020 aa protein sequence, with a putative rust resistance protein *Rp1-dp8* in *Brachypodium distachyon* (Figure 4, Table 1 and Table S10). The *Rp1-dp8-*like NLR gene was situated at 9.6 Mb in the Lo7 reference genome, and, in the Weining reference genome, the two respective NLR genes were situated at 18.9 and 19.1 Mb. 

## 4. Discussion

### 4.1. Evidence of Quantitative Resistance in the Germplasm

Most race-specific resistance (*R*) genes in plants are subject to classical gene-for-gene interactions with avirulence gene(s) in the pathogen, giving rise to effector-triggered immunity responses [38,70]. In turn, selection drives the emergence of virulent pathogen genotypes, permitting the evasion of R protein recognition, often termed the ‘arms-race’ [71,72]. As a result, monogenic inherited *R* genes exerting race-specific resistance under the gene-for-gene principle have been associated with short durability and loss of effect for disease control. In Canada, the wheat leaf rust pathogen *P. triticina* has been closely surveyed since the early 1900s, documenting, amongst others, the rapid loss of effectiveness by race-specific *R* genes such as *Lr1*, *Lr13*, *Lr14a* and *Lr26* [73,74,75]. Contrary to *R* genes conferring race-specific resistance, quantitative resistance conferring broad-spectrum resistance has been associated with enhanced durability [76,77]. Quantitative resistance, also referred to as partial resistance, is expressed as a susceptible infection type with reduced infection frequency and severity [78]. While major *R* genes exerting quantitative resistance have been identified, such as *Lr34* in wheat [79], quantitative resistance is often governed by several low-to-intermediate-effect quantitative trait loci (QTL) [80]. The potential additive effect, however, may permit a high level of resistance by the pyramiding of multiple QTLs, conferring quantitative resistance to leaf rust [81,82,83]. The adoption of marker-assisted selection (MAS) in modern plant breeding systems has aided in breeding for oligogenic traits, such as quantitative resistance [44,84]. In a large hybrid wheat breeding system, Beukert et al. [85] found that MAS constituted an efficient strategy for the introgression of leaf rust resistance QTLs. While quantitative resistance has been observed in inbred lines and population varieties of rye, no genes or QTLs have, to our knowledge, been deployed in commercial hybrid rye cultivars [6,86,87].

In the assayed hybrid rye breeding germplasm, we observed a subset of breeding lines in both parental populations (belonging to the assigned ‘P-R’ group), which demonstrated a moderate to high level of quantitative resistance. These breeding lines were characterized by the later occurrence of leaf rust and reduced disease progression compared to susceptible lines. In the non-restorer germplasm population, none of the quantitative resistant lines were found to harbor the resistant haplotype of the most leaf rust resistance-associated markers on chromosome arm 7RS. This finding could suggest that the quantitatively resistant lines do not carry the major *Pr* gene, instead either harboring rare variants of a large effect, or several common QTLs of low to intermediate effect [88]. In both cases, this would dramatically reduce the phenotypic variance explained and hence the statistical power of GWAS to infer marker linkage.

In the restorer population, however, several of the quantitatively resistant lines carried the resistance allele of the putative *Pr3*-associated marker on chromosome arm 1RS. If the causative gene is *Pr3*, this could be explained by the observation of *Prs* pathotypes virulent to *Pr3* in Germany by Roux et al. [35] in 2004. However, the strict conservation of the putative *Pr3*-associated marker amongst resistant or partially resistant lines indicates that the *R* gene remains largely effective towards a large fraction of current pathotypes in Northern Germany and Denmark. However, variation in copy number [89] or structural variation [90] of the putative *Pr3* gene may also give rise to the observed quantitative resistance in these lines. This could be the case for the quantitatively resistant non-restorer germplasm lines, potentially carrying a unique variant of the resistant locus on chromosome arm 7RS.

### 4.2. Discovery of a Novel Major Pr Gene on Rye Chromosome Arm 7RS in the Non-Restorer Germplasm Population

The use of genome-wide association studies (GWAS) has become a routine strategy for the mining of *R* genes in crop species [91,92]. Exploiting recent advances in rye genomic resources, we successfully identified a genomic region on the chromosome arm 7RS that was significantly associated with leaf rust resistance [39,40]. With no previous *Pr* gene identified on chromosome arm 7RS, we provisionally denote the new *Pr* gene discovered in this study *Pr6*, in accordance with the nomenclature presented by Wehling et al. [34].

While *Pr6* is a new finding in rye, several leaf rust *R* genes have been identified in wheat and barley chromosomal segments syntenic to the rye chromosome arm 7RS. During *Triticeae* speciation, a series of recurrent translocation events gave rise to major patterns of chromosomal rearrangements, disturbing the collinearity of orthologous chromosomes [93,94]. In barley, the chromosome arm 5HL, which is syntenic to the distal tip of chromosome arm 7RS, harbors three major leaf rust *R* genes: *Rph9*, *Rph9.z* and *Rph12* [95,96,97]. In wheat, the 4A, 5B and 5D chromosomes are syntenic to rye chromosome arm 7RS [40]. In total, ten major leaf rust *R* genes have been identified on wheat chromosomes syntenic to rye chromosome arm 7RS, comprising two on 4AL (*Lr28*, *Lr30*), three on 5BS (*LrK1*, *Lr52*), two on 5BL (*Lr18*, *Lr*), three on 5DS (*Lr57*, *Lr70*, *Lr76*) and two on the 5DL (*Lr1*, *LrSyn137*) chromosome [98].

### 4.3. Discovery of a Putative Pr3 Gene in the Restorer Population

While the majority of resistant lines in the assayed germplasm belonged to the restorer population, no genomic region significantly associated with resistance could be identified by GWAS on the parental population alone, including the *Pr6* locus. However, several non-significant peaks were identified that could potentially correspond to both known, such as *Pr6*, and novel *Pr* genes [34,35]. GWAS used BLUE estimated resistance values across all trial locations on the entire population, which resulted in a resistance-associated SNP marker physically co-localizing with molecular markers delimiting *Pr3* on chromosome arm 1RS [35]. Intriguingly, the resistant allele of the putative *Pr3*-associated marker was strictly confined to the restorer population. While this indicates that the putative *Pr3* locus is prevalent amongst resistant and partially resistant restorer lines, several did not carry the resistant allele. In conjunction with the finding of several non-significant, low-to-intermediate-effect QTLs in GWAS on the restorer population, this suggests the presence of additional, less prevalent *R* genes or low-to-intermediate-effect QTLs.

The ability of GWAS to establish an association between a marker and trait of interest is influenced by several biological factors [88]. In a recent population study by Vendelbo et al. [50] on the assayed germplasm, the population genetic characteristics diverged considerably, with the restorer population showing high genetic diversity, effective population size and low linkage disequilibrium The higher frequency of effective recombination events will in turn cause a more rapid decay in linkage; hence, influencing the extent of linkage between non-functional markers and the *R* gene of interest needed in GWAS to establish a significant association [88,99]. In heterogeneous cross-fertilizing crop species, the rate of decay is often rapid compared to self-pollinated species [100]. Additionally, the existence of several less prevalent *Pr* genes and low-to-intermediate-effect QTLs in various combinations would, in addition to the low sample size, further reduce the phenotypic variance explained by genes needed for GWAS to establish a significant association [92]. The potential existence of a more complex genetic architecture underlying leaf rust resistance in the restorer population is supported by the high level and diverse spectra of leaf rust resistance, discovery of several low-to-intermediate-effect QTLs and the absence of the putative *Pr3* locus in some resistant lines.

### 4.4. Pr6, a Potential Ortholog to Wheat Leaf Rust Resistance Gene Lr1 on Rye Chromosome Arm 7RS

Enabled by recent advances in rye genomic resources, we investigated whether the identified distal region of chromosome arm 7RS associated with *Pr6* harbored NLRs resembling known leaf rust *R* genes. In plants, NLR genes have been observed to accumulate in large clusters at recombination hotspots in the subtelomeric region, contributing to the rapid generation of novel genetic variation in NLR genes [99,101]. To detangle the NLR diversity, we conducted a phylogenetic analysis using the conserved NB-ARC domain, including cloned cereal rust genes as a reference [102,103]. The analysis led to the identification of a large clade of paralogous NLR genes showing a close evolutionary relationship and protein sequence similarity with wheat leaf rust *R* gene *Lr1*. In wheat, *Lr1* has been mapped to the 5D chromosome syntenic to rye chromosome arm 7RS [40,104]. Initial fine mapping of *Lr1* revealed a close linkage of the gene to a molecular marker, *Xpsr567* [105,106]. Intriguingly, a successive study mapped the *Xpsr567* marker to the distal region of rye chromosome arm 7RS [107]. These findings suggest that the novel leaf rust *R* gene *Pr6* could potentially be a rye ortholog to *Lr1*. A similar observation has been made on powdery mildew by Hurni et al. [108], who demonstrated that the powdery mildew resistance genes *Pm8* on rye 1RS and *Pm3* on the syntenic chromosome arm 1AS in wheat are orthologous genes. Despite an independent evolution since the species diverged 7 million years ago, the orthologous powdery mildew *R* genes retained a similar resistance function. *Pr6* in rye and *Lr1* in wheat could be another such example. However, the in silico study presented here only constitutes a preliminary finding of a potential co-evolution between a wheat and rye leaf rust *R* gene. In order to investigate whether *Pr6* constitutes an *Lr1* ortholog, identification of the causative gene, e.g., by resistance gene enrichment sequencing (RenSeq) analysis, followed by the transformation of a susceptible non-restorer germplasm line, would be required [109].

## 5. Conclusions

In contrast to previously investigated hybrid rye breeding germplasms, the assayed Gülzow germplasm displayed a high level of qualitative as well as quantitative resistance to leaf rust, providing opportunities for the development of leaf-rust-resistant rye hybrids. By performing GWAS on 261,406 informative SNP markers, we identified a putative *Pr3* gene confined to the restorer population and a novel *Pr* gene on chromosome arm 7RS, provisionally denoted *Pr6*, confined to the non-restorer germplasm population. Using recent advances in rye genomic resources, we identified a large cluster of *Lr1*-like NLR genes residing in close proximity to *Pr6*. With wheat leaf rust *R* gene *Lr1* situated on 5D homologous to rye 7RS, our finding suggests that *Pr6* could potentially be a rye ortholog to *Lr1*. Despite the discovery of two *Pr* genes, our findings show that GWAS was impeded by several co-existing biological factors. To unveil the genetics underlying leaf rust resistance in the genetically diverse restorer population, we recommend developing bi- and multiparent mapping populations for the restorer, in addition to increasing the sample size [110,111]. This approach would equally permit the concurrent investigation of the genetics underlying leaf rust resistance in the quantitatively resistant lines, constituting a valuable genetic resource for enhancing the resistance durability [83]. As *R* genes in rye can be introgressed into wheat by chromosomal translocation and substitution lines, gene mining in rye serves a dual purpose, accentuating the relevance of studies in rye [60,112].

## Figures and Tables

**Table 1:** Characteristics of nucleotide-binding leucine-rich repeat (NLR) genes residing in leaf resistance-associated region on rye (*Secale cereale* L.) chromosome arm 7RS in the reference genomes Lo7 and Weining showing similarity with known leaf rust resistance genes.

Reference Genome					Coding Sequence (aa)	BlastP				
	NLR ID	Position (Mbp)	Gene Length (bp)	Predicted Protein Sequence Length (aa)		Hit	Species	Alignment Length (aa)	Identity (%)	Gaps (%)
Lo7	Lo7_chr7R_nlr_9	2.37	3294	1098	1408	*Lr1 disease protein*	*Triticum aestivum*	1183	84.60	4.18
	Lo7_chr7R_nlr_10	2.41	3141	1047	1326	*Lr1 disease protein*	*Triticum aestivum*	1270	80.48	6.31
	Lo7_chr7R_nlr_12	2.80	3258	1086	1387	*Lr1 disease protein*	*Triticum aestivum*	1341	80.67	5.44
	Lo7_chr7R_nlr_13	2.81	3246	1082	1429	*Lr1 disease protein*	*Triticum aestivum*	1195	81.99	4.00
	Lo7_chr7R_nlr_15	2.87	3207	1068	1438	*Lr1 disease protein*	*Triticum aestivum*	1195	84.69	3.75
	Lo7_chr7R_nlr_33	9.57	2294	526	-	*Putative rust resistance protein Rp1-dp8*	*Brachypodium distachyon*	350	62.90	17.74
Weining	Wei_chr7R_nlr_15	12.21	3687	1075	-	*Lr1 disease protein*	*Triticum aestivum*	1064	83.72	4.29
	Wei_chr7R_nlr_17	12.35	3207	1069	-	*Lr1 disease protein*	*Triticum aestivum*	1054	85.41	3.88
	Wei_chr7R_nlr_18	12.46	6438	1730	-	*Lr1 disease protein*	*Triticum aestivum*	1113	86.03	3.87
	Wei_chr7R_nlr_20	12.53	3294	1098	-	*Lr1 disease protein*	*Triticum aestivum*	1078	84.32	3.87
	Wei_chr7R_nlr_27	14.41	1392	464	-	*Lr1 disease protein*	*Triticum aestivum*	464	81.66	3.41
	Wei_chr7R_nlr_29	14.52	11,906	1044	-	*Lr1 disease protein*	*Triticum aestivum*	915	82.43	5.75
	Wei_chr7R_nlr_31	18.91	2054	500	-	*Putative rust resistance protein Rp1-dp8*	*Brachypodium distachyon*	534	54.68	25.72
	Wei_chr7R_nlr_32	19.09	2053	440	-	*Putative rust resistance protein Rp1-dp8*	*Brachypodium distachyon*	366	59.95	18.11

## Data Availability

The datasets presented in this study can be found in the Supplementary Materials.

## Supplementary Materials

The following are available online at https://www.mdpi.com/article/10.3390/cells11010064/s1, Figure S1: Manhattan plot for genome-wide association study (GWAS) using BLINK method of leaf rust disease resistance in an entire hybrid rye (*Secale cereale* L.) breeding germplasm (‘All’), or parental populations, restorer (‘R’, *n* = 92) and non-restorer germplasm (‘NRG’, *n* = 88) using 261,406 informative SNP markers, Table S1: 1–9 scoring scale for leaf rust disease severity in rye (*Secale cereale* L.), Table S2: Leaf rust disease severity of top-yielding hybrid rye (*Secale cereale* L.), hybrid rye population mixtures and population varieties tested in the Danish official trials in 2019 and in the German list of recommended varieties in 2021, Table S3: Leaf rust disease severity score (1–9) of 180 Nordic Seed hybrid rye (*Secale cereale* L.) elite breeding lines across four field trials in Denmark and Northern Germany in 2019 and 2020, Table S4: Leaf rust disease severity score (1–9) of two hybrid rye (*Secale cereale* L.) cultivars tested in six replicates at four field trials in Denmark and Northern Germany in 2019 and 2020, Table S5: Distribution of 180 Nordic Seed hybrid rye (*Secalae cereale* L.) elite breeding lines in four leaf rust resistance groups based on area under disease progression curve and disease progression pattern, Table S6: Metrics and genotype of top-most leaf rust resistance associated markers in Nordic Seed hybrid rye (*Secale cereale* L.) elite breeding lines, Table S7: Variance components and plot broad sense heritability of leaf rust resistance in 180 Nordic Seed hybrid rye (*Secale cereale* L.) inbred lines evaluated at four field trials in Denmark and Northern Germany. The material comprised of 88 non-restorer germplasm lines (N), 92 restorer lines (R), Table S8: Mapping position of leaf rust resistance-associated markers on chromosome 7RS on Lo7 reference genome to Weining reference genome, Table S9: Annotated nucleotide-binding leucine-rich repeat (NLR) genes residing in a leaf rust resistance-associated region on chromosome arm 7RS in Nordic Seed hybrid rye (*Secale cereale* L.) elite breeding germplasm in Lo7 and Weining reference genomes, Table S10: In silico characterization of nucleotide-binding leucine-rich repeat genes residing in leaf rust resistance-associated region on rye (*Secale cereale* L.) chromosome arm 7RS in Lo7 and Weining reference genomes, Supplementary Material S1: NB-ARC sequence of nucleotide-binding leucine-rich repeat (NLR) genes residing in leaf rust resistance-associated region on rye (*Secale cereale* L.) chromosome arm 7RS in Lo7 and Weining reference genomes, Supplementary Material S2: Genotype of 261,406 Informative single-nucleotide polymorphism (SNP) markers in 180 Nordic Seed hybrid rye (*Secale cereale* L.) elite breeding lines.
