# Supplementary material for: Discovery of a Novel Leaf Rust (Puccinia recondita) Resistance Gene in Rye (Secale cereale L.) Using Association Genomics
Source: Cells. 2021 Dec 27;11(1):64. doi: 10.3390/cells11010064 (PMC8750363; doi:10.3390/cells11010064)
Supplement: Supplementary file 1 [file cells-11-00064-s001.zip › Supplementary_materials/Supplementary material captions.pdf]

Supplementary material

Supplementary Table S1: 1-9 scoring scale for leaf rust disease in rye (*Secale cereale* L.)

Supplementary Table S2: Leaf rust disease severity of top-yielding hybrid rye (*Secale cereale* L.) and hybrid rye-population mixtures tested in the Danish official trials in 2019 across nine locations in Denmark (Sortinfo, 2021)

Supplementary Table S3: Leaf rust disease severity score (1-9) of 180 Nordic Seed hybrid rye (*Secale cereale* L.) elite breeding lines across four locations in Denmark and Northern Germany in 2019 and 2020. Lines were grouped according to their disease progression profile at the untreated trial site Gylling in 2019, and their area under dissease progression curve (AUDPC). Groups were resistant ('R'), partial resistant ('P-R'), partial susceptible ('P-S'), and susceptible ('S').

Supplementary Table S4: Leaf rust disease severity score (1-9) of two hybrid rye (*Secale cereale* L.) cultivars tested in six replicates at four field trial locations in Denmark and Northern germany in 2019 and 2020.

Supplementary Table S5:

a. Mean leaf rust disease severity (1-9) and standard deviation for each assigned resistance spectrum groups (R: resistant, P-R: partial resistant, P-S: partial susceptible, S: susceptible) across 180 Nordic Seed hybrid rye (*Secale cereale* L.) elite breeding lines phenotyped at four field trial locations in Denmark and Northern germany in 2019 and 2020.
b. Distribution of 180 Nordic Seed hybrid rye (*Secale cereale* L.) elite breeding restorer (R) and non-restorer germplasm (NRG) lines in four leaf rust resistance spectrum groups. Resistant ('R'), partial resistant (P-R), partial susceptible (P-S), and susceptible (S).

Supplementary Table S6: Top 1000 leaf rust resistance associated 600K SNP markers from Genome-wide association study on the entire Nordic Seed hybrid rye (*Secale cereale* L.) elite breeding germplasm (n = 180), or parental populations; restorer (n = 92), non-restorer germplasm (n = 88) using BLUP estimated resistance value on 2018-2019 field trial phenotyping data.

Supplementary Table S7

a. Top leaf rust disease resistance associated 600K array SNP markers in Nordic Seed hybrid rye (*Secale cereale* L.) elite breeding germplasm (n = 180) phenotyped at four field trial locations in Northern Germany and Denmark in 2019 and 2020 identified by GWAS using BLINK method
b. Genotype of top leaf rust disease resistance associated 600K array SNP markers in Nordic Seed hybrid rye (*Secale cereale* L.) elite breeding germplasm (n=180) identified in GWAS using BLINK method. Germplasm comprised of 92 restorer (R) and 88 non-restorer germplasm (N) lines, grouped into four categories based on their resistance response towards leaf rust resistance, 'R' resistant, 'P-R' partial resistant, 'P-S' partial susceptible, and 'S' susceptible.

Supplementary Table S8:

a. Informative 600K SNP array markers in region on chromosome arm 7RS in the 'Lo7' reference genome associated with leaf rust resistance in 180 Nordic Seed hybrid rye (*Secale cereale* L.) elite breeding lines
b. Mapping of 600K SNP array markers associated with leaf rust resistance in 180 Nordic Seed hybrid rye (*Secale cereale* L.) elite breeding lines on chromosome arm 7RS in the 'Lo7' reference genome to 'Weining' rye reference genome using NCBI blastn function

Supplementary Table S9: Annotated nucleotide-binding leucine rich repeat (NLR) genes residing in a leaf rust resistance associated block on chromosome arm 7RS in Nordic Seed hybrid rye (*Secale cereale* L.) elite breeding germplasm (n = 180) in

a. 'Lo7' reference genome
b. 'Weining' reference genome

Supplementary Table S10:

a. Characteristics of potential candidate leaf rust resistance nucleotide binding leucine rich repeat genes residing in leaf resistance associated block on rye (*Secale cereale* L.) chromosome arm 7RS
b. Pairwise multiple sequence alignment identity of potential candidate leaf rust resistance nucleotide binding leucine rich repeat genes residing in leaf resistance associated block on rye (*Secale cereale* L.) chromosome arm 7RS

Supplementary Material 1: NB-ARC sequence of nucleotide-binding leucine-rich repeat (NLR) genes residing in leaf rust resistance associated block on chromosome arm 7RS in 'Lo7' and 'Weining' reference genome

Supplementary Material 2: Genotype of 261,406 Informative single-nucleotide polymorphism (SNP) markers in 180 Nordic Seed hybrid rye (*Secale cereale* L.) elite breeding lines.

Supplementary Figure S1: Manhattan plot for genome wide association study (GWAS) using BLINK method of leaf rust disease resistance in an entire hybrid rye (*Secale cereale* L.) breeding germplasm ('All'), or parental populations, restorer ('R', n = 92) and non-restorer germplasm ('NRG', n= 88) using 261,406 informative SNP markers including QQ plots. Phenotypic input for the analysis were a resistance value estimated per breeding line across the four trial locations in 2019 and 2020 in Denmark and Northern Germany using a linear regression model.
