# Supplementary material for: Discovery of a Novel Leaf Rust (Puccinia recondita) Resistance Gene in Rye (Secale cereale L.) Using Association Genomics
Source: Cells. 2021 Dec 27;11(1):64. doi: 10.3390/cells11010064 (PMC8750363; doi:10.3390/cells11010064)
Supplement: Supplementary file 1 [file cells-11-00064-s001.zip › Supplementary_materials/Supplementary Table S1.pdf]

Supplementary Table S1: 1-9 scoring scale for leaf rust disease severity in rye (*Secale cereale* L.)

**Group**	**Score**	**Phenotype**	**Leaf Coverage (%)**	**Incidence (%)**
Resistant	1	No disease	0-0.01	0
	2	Few colonies/pustules/spots per plant	0.1	1-49
Partially Resistant	3	Few colonies/pustules/spots per tiller	0.5	51-100
	4	Several colonies/pustules/spots on lower leaves: < 10 % coverage	1	100
Partially Susceptible	5	Lower leaves: 10-25 % coverage	5	100
	6	Upper leaves: <10% coverage, lower leaves: 25-50 % coverage	10	100
Susceptible	7	Upper leaves: <25% coverage, lower leaves: 50-75 % coverage	25	100
	8	Half of leaves dead with lower leaves: 75-95 % coverage	50	100
	9	No green	75-100	100
